# LKB1 Inhibits Breast Cancer Partially through Repressing the Hedgehog Signaling Pathway

**DOI:** 10.1371/journal.pone.0067431

**Published:** 2013-07-04

**Authors:** Zhigang Zhuang, Kai Wang, Xiaolin Cheng, Xueying Qu, Beiqi Jiang, Zhengdong Li, Jianming Luo, Zhiming Shao, Tao Duan

**Affiliations:** 1 Department of Breast Surgery, Shanghai First Maternity and Infant Hospital, Tongji University School of Medicine, Shanghai, China; 2 Breast Cancer Institute, Cancer Hospital, Shanghai Medical College, Fudan University, Shanghai, China; Wayne State University School of Medicine, United States of America

## Abstract

Constitutive activation of the Hedgehog (Hh) signaling pathway has been implicated in the development of many human malignancies. Hh targets, such as Patched (PTCH), smoothened (SMO), Sonic hedgehog (SHH) and glioma-associated oncogene homologue 1 (GLI1), are markers of Hh signaling activation and expressed in most Hh-associated tumors. The protein kinase LKB1 has been shown to slow proliferation and induce cell-cycle arrest in many cell lines. In this study, we observed that activated LKB1 decreased the expression of factors related to Hh reporter activity in MDA-MB-231 breast cancer cells, including of SMO, SHH and GLI1. In contrast, LKB1 siRNA increased the expression of these target genes. The same results were shown to inhibit the Hh factors Sufu and Hip. Furthermore, we also observed negative correlation between LKB1 and glioma-associated oncogene homologue 1 (GLI1) in three breast cancer cell lines. Meanwhile, LKB1 siRNA rescued the inhibition of cell growth by 3-Keto-N-(aminoethyl-N'-aminocaproyldihydrocinnamoyl) cyclopamine (KAAD-cyclopamine), an antagonist of the Hh element SMO, which suggests that LKB1 acts as the downstream of SMO. In vivo, LKB1 siRNA increased tumor growth in the mammary fat pad, and the expression levels of Hh displayed similar results in vitro. Overexpression of the LKB1 protein in human breast cancers is associated with the expression of Hh. We found that breast carcinomas with detectable Hh had weak or undetectable expression of LKB1, whereas tumors that expressed high levels of LKB1 had undetectable Hh signaling. In this study, we find that LKB1 are negatively correlated with the expression of Hh related transcription factors. These findings suggest that LKB1 may inhibit tumorigenesis by regulating Hh signaling in certain cancers.

## Introduction

The liver kinase B1 (LKB1) is a serine/threonine protein kinase encoded by the tumor-suppressor gene, STK11 (locus on 19p13.3). It is mutated in autosomal-dominant inherited Peutz-Jeghers syndrome (PJS), a disease characterized by predisposition to gastrointestinal polyposis, increased risk of benign and malignant tumors in multiple tissues and mucocutaneous pigmentation [1]. Liver kinase 1 (LKB1) is emerging as a multifunctional protein, acting as a key metabolic enzyme, regulator of cell polarity, and transcription factor. Recent studies have demonstrated that LKB1 regulates cell growth, cell proliferation and cell survival in response to different stresses. Altered LKB1 expression has been linked with various cancers development and growth [2]. Our previous study showed that overexpression of LKB1 protein inhibits MDA-MB-435 cancer cells proliferation, migration and invasion. Moreover, low expression of the LKB1 protein in human breast cancer is significantly associated with a shorter survival [3], [4]. These results clearly suggested that LKB1 might exert tumor inhibitory effects on human breast cancer. However, the precise molecular mechanisms underlying these anti-cancer actions are still unclear.

It is well demonstrated that Hedgehog (Hh) signaling pathway controls a variety of developmental processes, including cell proliferation, differentiation and survival [5]. Dysregulation of the Hh pathway results in cellular hyperproliferation and contributes to the formation and progression of human cancers, including lung, breast, pancreatic and prostate cancers [6]–[8]. Hh signal transduction is started by the binding of the Hh ligand to its receptor Patched (PTCH). In the absence of the Hh, PTCH represses Hh-pathway activity by inhibiting the seven transmembrane receptors, smoothened (SMO). Upon Hh binding, the inhibitory function of PTCH on SMO is abolished, resulting in SMO activation. Then SMO is able to transduce the signaling cascade through the glioma-associated oncogene homologue 1 (GLI1) transcriptional activator form, and regulate the expression of Hh target genes including GLI1 and PTCH, making these genes markers for Hh-pathway activation [9], [10]. Recently, the functional roles of Hh signaling pathway in breast cancer has been widely studied. However, the correction between LKB1 and Hh signaling pathway has not been elucidated.

In this study, we determined that LKB1 antagonizes Hh protein expression in MDA-MB-231 cells and that endogenous LKB1 has a negative effect on Hh activity in human breast cancer cells. In addition, we found that the expression of LKB1 is markedly decreased or undetected in breast cancers with activated Hh signaling. These results are consistent with the general function of LKB1 for attenuating proliferation and inducing cell apoptosis. Our findings also provide the first evidence that suggests the negative regulation of the Hh pathway by LKB1.

## Materials and Methods

### Construction of the LKB1 Expression Plasmid

The expression plasmid pcDNA3.1/LKB1 myc, which contains the wild-type LKB1 coding sequence, was constructed using PCR. A DNA fragment amplified using PCR with the LKB1 primer (5′-GATGAATTCGGGTCCAGCATGGAGGTGGTGGAC-3′) and LKB2 primer (5′-GATGAATTCTTAGAGGTCTTCTTCTGAGATGAGCTTCTGCTCCTGCTGCTTGCAGGCCGA-3′) was cloned into the *Eco*RI site of the *pcDNA3* vector. The clones with the correct orientation were selected, and their sequences were verified.

### Cell Culture and Transfections

The breast cancer cell line MDA-MB-231, MDA-MB-436 and MDA-MB-453 were grown in DMEM supplemented with 10% (v/v) fetal bovine serum and antibiotics (100 units/mL penicillin and 100 µg/mL streptomycin). The cells were cultured as monolayers in a 95% air and 5% CO_2_ water-saturated atmosphere. MDA-MB-231 cells, a LKB1 negative cell line shown by RT-PCR and western blot analysis, were transfected with the pcDNA3.1/LKB1 expression vector using the LipofectAMINE method (Life Technologies; Rockville, MD). The transfected cells were subjected to 1 mg/mL G418 selection. Several G418-resistant clones were tested for the integration of the LKB1 expression vector into the transfected cells. The LKB1-transfected cells expressing increased LKB1 mRNA and protein used in our previous studies [3], [4]were used in this project.

### siRNA transfections

Cells were seeded into 6-well plates and cultured for 4 h. Transient siRNA transfections were performed using Hiperfect reagent (Qiagen; FRA, Germany).The siRNAs targeting LKB1 were purchased from Dharmacon (ON-TARGET plus SMART Pool Human STK11, L-005035-00) following the manufacturer’s recommendations with some modifications. The media was changed every 24 h, and the cells were harvested at 72 h post-transfection to analyze knockdown efficiency. For reporter assays, cells were transfected with pGL3 vectors after 48 h of siRNA transfection and harvested after 24 h at 37°C.

### Isolation of RNA and RT-PCR

Total RNA was extracted with TRIzol reagent (Life Technologies). RNA (0.8 µg) was used in the reverse transcription reaction. The standard random priming method with Moloney murine leukemia virus reverse transcriptase (Promega; Madison, WI) and RNase inhibitor (Promega) was used to obtain 20 µL of cDNA. Reactions were run on a DNA Engine Opticon 2 System (Bio-Rad, Hercules, CA, USA) using SYBR Premix Ex Taq II (Takara, Otsu, Japan). Cycling conditions were performed as previously described. The PCR products were electrophoresed on a 1.5% agarose gel and imaged on a ChemiImage 5500 Imaging System (Alpha Innotech; San Leandro, CA). Densitometry of the images was performed using NIH Image version 1.62 software. The specific primers and their annealing temperatures are listed in Table 1.

**Table 1:** Primer sequences and annealing temperatures used in RT-PCR.

Shh	F 5′GACTCAGAGGTGTAAGGACAAGTT3’ R 5′CTCGGTCACCCGCAGTTT3’	85 bp	59°C
Smo	F 5′CAACCTCTTTGCGTTTCCTT3’	154 bp	59°C
	R 5′ACTCACTGCTCCTATCCCACTC3’		
Ptch	F 5′CAGGCAGCGGTAGTAGTGG3’	193 bp	59°C
	R 5′TGTAGCGGGTATTGTCGTGT3’		
Sufu	F 5′GTTGGAGGATTTAGAAGATTTGAC3’	132 bp	59°C
	R 5′CCAGGCTAGTGTAGCGGAC3’		
Hip	F 5′TTCAGTAATGGTCCTTTGGTTG3’	164 bp	59°C
	R 5′CTAGTGCCGAGACAGAGTGGT3’		
LKB1	F 5′CGGCAAGGTGAAGGAA3’	141 bp	59°C
	R 5′ACGCCCAGGTCGGAGAT3’		
GAPDH	F 5′GGGAAACTGTGGCGTGAT3’	299 bp	56°C
	R 5′GAGTGGGTGTCGCTGTTGA3’		

### Western Blotting

Cells were washed twice with cold PBS, harvested, and lysed by sonication in buffer (20 mM imidazole-HCl, 2 mM EGTA, 2 mM EDTA [pH 7.0], 1 mM benzamidine, 1 mMPMSF, 1% NP40, 5 lg/ml of leupeptin, and 5 lg/ml of aprotinin). The lysates were centrifuged (1,000×*g* 10 min,4°C), and protein concentrations of the supernatant were determined. Proteins (20 µg) were separated on 10% SDS-PAGE gels, electroblotted onto an Immobilon-P membrane (Millipore, Bedford, MA). Western blots using murine monoclonal antibodies to LKB1, Hh-related factors (Oncogene Research Products; Cambridge, MA) and LKB1 polyclonal antibodies (Upstate Biotechnology; Lake Placid, NY) were performed following standard protocols. Blot quantitation was performed using a Molecular Dynamics laser densitometer (Model PSD; Mount Holly, NJ) and ImageQuant version 1 software.

### Luciferase Reporter Assays

To measure GLI-mediated Hh transcriptional activity, the luciferase reporter constructs, wild-type GLI binding site (Gliwt Luc) or mutant GLI binding site (Glimut Luc) plasmids and a human GLI1 expression vector (pcDNA3.1-GLI1) were co-transfected into cells in 24-well plate. The Renilla luciferase pRL-TK plasmid (Promega, Madison, WI), whose expression is driven by the housekeeping thymidine kinase gene promoter, was co-transfected to normalize for transfection efficiency. All transfection experiments were performed using the Lipofectamine2000 (Invitrogen) in accordance with the manufacturer’s instructions. After 24 h cells were lysed and luciferase assays were performed as described previously. Results are expressed as fold induction, which is the ratio of luciferase activity induced in GLI-transfected cells relative to basal luciferase activity in control transfected MDA-MB-231, MDA-MB-453,MDA-MB-436 cells. All experiments were performed in triplicate; means and standard errors were calculated using Student’s t-test.

### Flow Cytometry Analysis

A total of 1×10^6^ MDA-MB-231 and LKB1-transfected MDA-MB-231 cells were harvested after 48 h of cyclopamine treatment in four different concentrations (0 mol/L, 0.5×10^−6^ mol/L, 10×10^−6^ mol/L and 20×10^−6^ mol/L). The cells were washed twice with cold PBS, resuspended in 2 ml of 70% ethanol and maintained at 4°C overnight. The cells were rinsed twice with PBS and incubated with 100 µl RNase (10 mg/ml). Finally, the cells were stained with BrdU (for cell cycle analysis) or propidium iodide (for apoptosis analysis). Distribution of the cell cycle and rate of apoptosis were determined using flow cytometry (Becton Dickinson; Franklin Lakes, USA).

### In vivo Animal Studies

Female athymic BALB/c nu/nu 4-to-6-week-old mice were obtained from the Shanghai Institute of Material Medical, Chinese Academy of Sciences and housed in laminar flow cabinets under specific pathogen-free conditions. The Shanghai Medical Experimental Animal Care Committee approved the study protocol. The tumorigenicity and spontaneous metastatic capability of the cell lines were determined by injection into the mammary fat pad. A total 1×10^6^ cells in 0.1 mL of culture medium were inoculated into the anesthetized mouse. Animals were divided into three groups: MDA-MB-231, MDA-MB-231/vector, MDA-MB-231/siRNA LKB1+ cyclopamine and MDA-MB-231/siRNALKB1. Each group contained six mice. Animals were monitored every 2 days for up to 6 weeks for tumor growth and general health. Tumor volumes were calculated from two tumor diameter measurements using a vernier caliper: tumor volume = L × W × 1/2W. at 7-day intervals. Animals were killed and autopsied at 6 weeks after inoculation.

### Human Breast Cancer Sample Collection and Immunohistochemical Detection of LKB1 and Hh Signaling Molecules

Seventy-five patients with local breast carcinomas from the Shanghai First Maternity and Infant Hospital between March 2008 and March 2011 were included. The collection of tumor specimens and clinical and pathological information was reviewed and approved by the human research committees of the Cancer Hospital and informed consent was obtained from each patient. The tissues were taken as distant as possible during routine surgery and none of the adjacent normal tissues contained visible tumor contamination by histological analysis. Tissue sections (5 µm thick) were deparaffinized and dehydrated, followed by incubating the sections in a 10 mM citrate buffer solution (pH 6.0) in a microwave for 10 min for antigen retrieval. Then the tissue sections were immersed in 3% H_2_O_2_ in methanol for 10 min in order to quench endogenous peroxidase activity. After blocking the non-specific binding sites with 1% horse serum albumin, the sections were incubated with specific primary antibody for 1 h and a biotinylated mouse anti-rabbit antibody (ABC kit; Vector Laboratories) for 30 min. Evaluation of immunohistochemical staining using the Sinicrope scoring method was used to evaluate the intensity of the immunohistochemical staining and proportion of the stained epithelial cells. The staining intensity was classified as follows: (1) weak, (2) moderate, and (3) strong. The positive cells were quantified as a percentage of the total number of epithelial cells and assigned to one of five categories (0, <5%; 1, 5–25%; 2, 26–50%; 3, 51–75%; and 4, >75%). The percentage of positive tumor cells and staining intensities were multiplied to generate the immunoreactive score (IS) for each specimen. All immunostained tissue sections were semi-quantitatively rated on a four-grade scale (–, +, ++, and +++) for statistical analysis.

### Statistical Analyses

Data are represented as the mean ± SEM of at least two independent experiments and were analyzed using *t*-tests or one-way analysis of variance (ANOVA) followed by Tukey’s post-test for multiple comparisons to determine significant differences between groups (denoted by stars or different letters, respectively) using GraphPad Prism analysis software. A P value less than 0.05 was considered statistically significant.

## Results

### Overexpression or Knockdown of LKB1 in MDA-MB-231 Cells

**Figure 1:**
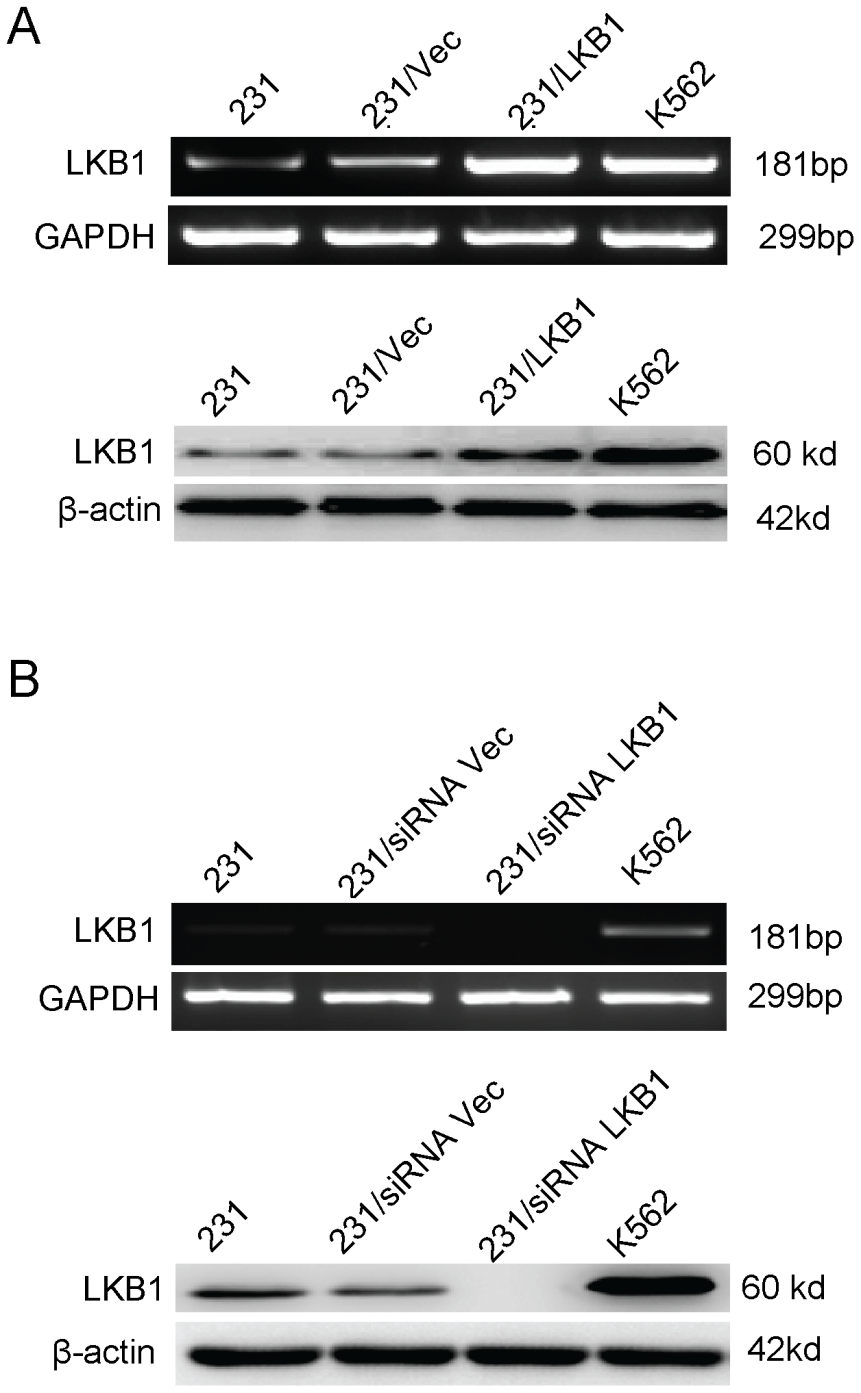
LKB1 expression in the breast cancer cell line MDA-MB-231 using RT-PCR and Western blot. (**A**) mRNA and protein expression of LKB1 in the MDA-MB-231, MDA-MB-231/vecto, MDA-MB-231/LKB1 and control (K562); (**B**) mRNA and protein expression of LKB1 in the MDA-MB-231, MDA-MB-231/siRNA vector, MDA-MB-231/siRNA LKB1 and control (K562).

To investigate the possible correlation of LKB1 with Hg signaling pathway, we firstly transfected an LKB1 expression vector into MDA-MB-231 cells and generated stable LKB1-overexpression cells. As shown in the Figure 1A, the mRNA and protein expression levels of LKB1 were significantly increased when compared with their expression in vehicle control using RT-PCR and Western blot analysis. Similarly, we silenced LKB1 expression using RNA interference. As we expected, transfection of siRNA targeting LKB1 resulted in a 90% reduction of LKB1 mRNA expression in MDA-MB-231 cells comparison with control siRNA (Figure 1B).

### Effects of Overexpression or Knockdown of LKB1 on the Hh Signaling Molecules in MDA-MB-231 Cells

**Figure 2:**
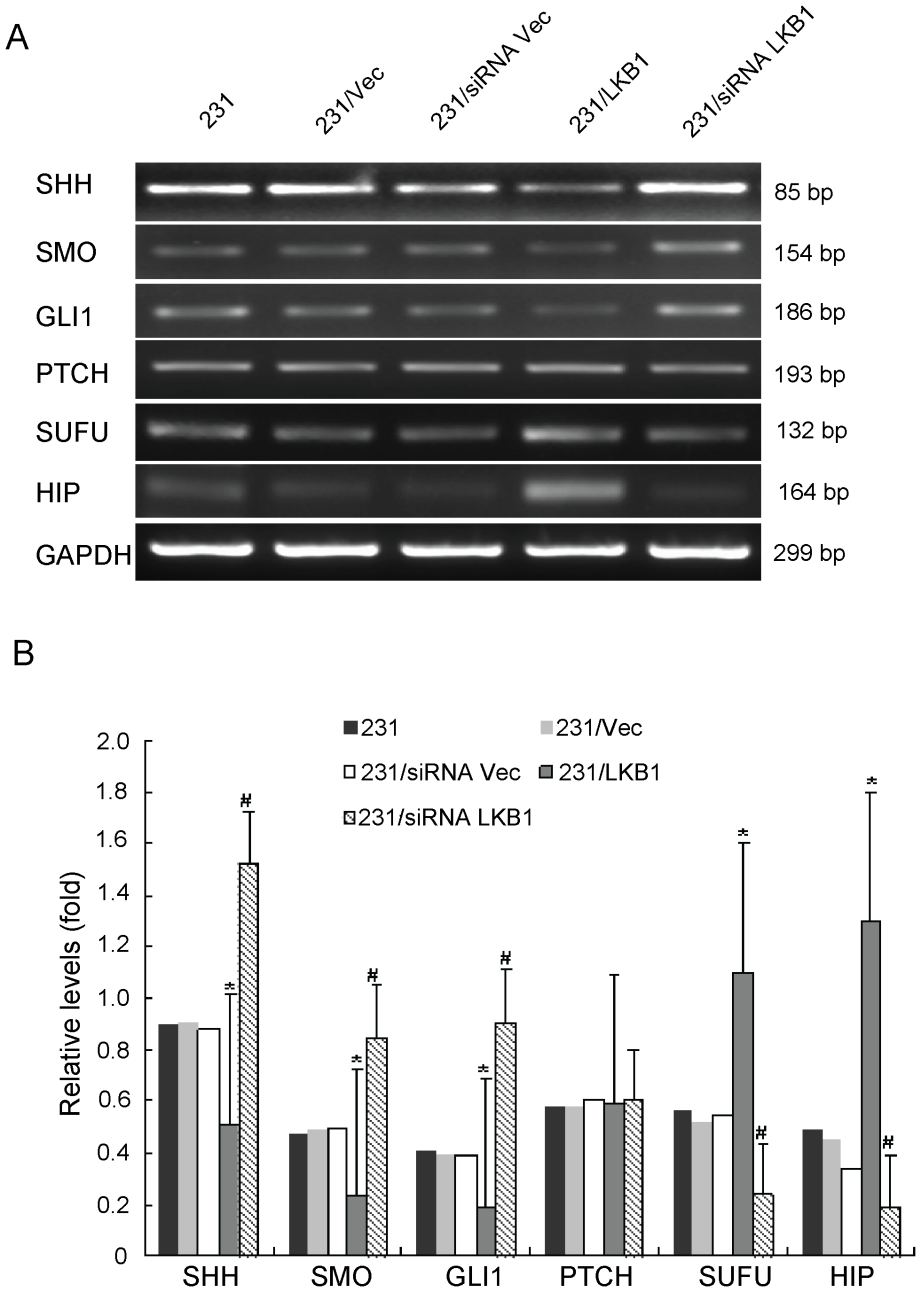
Analysis of effector molecules in Hh. (**A**)The relative levels of mRNA for SHH, SMO, GLI1, PTCH, SUFU, and HIP, which were detected by RT-PCR in MDA-MB-231, MDA-MB-231/vector, MDA-MB-231/siRNA vector, MDA-MB-231/LKB1, and MDA-MB-231/siRNA LKB1 cells. (**B**) Representative quantitation from three independent experiments. The values of MDA-MB-231 are expressed relative to the respective controls (GAPDH), which were given an arbitrary value of 1. Bars, SE. *p<0.05, #p<0.05.

**Figure 3:**
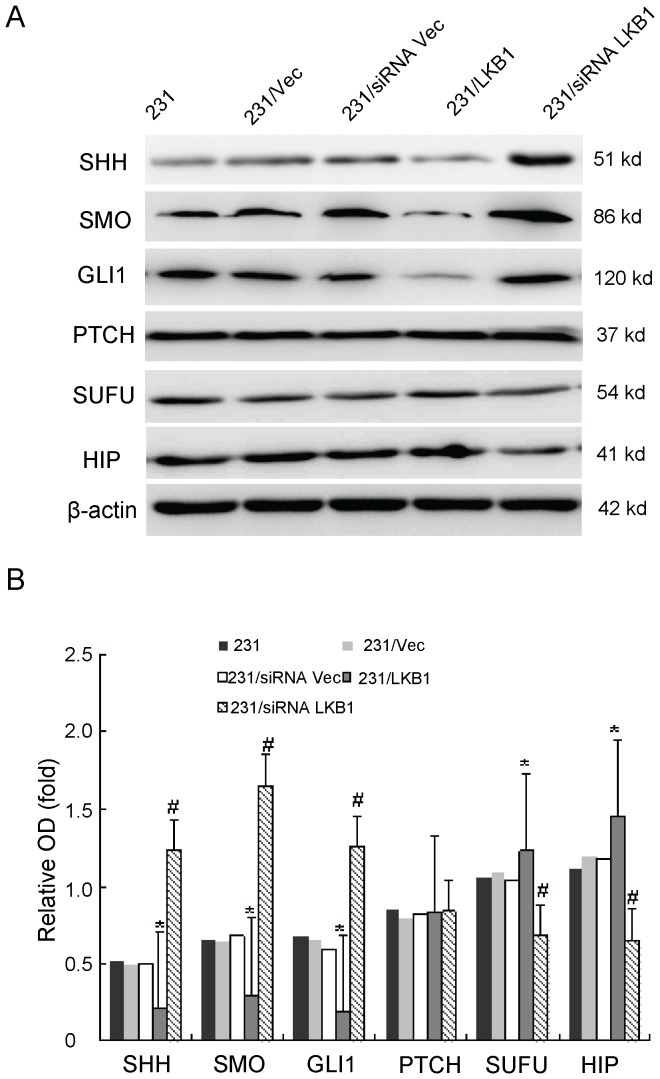
Analysis of effector molecules in Hh. (**A)**The relative protein levels of SHH, SMO, GLI1, PTCH, SUFU, and HIP, which were detected by Western blotting in MDA-MB-231, MDA-MB-231/vector, MDA-MB-231/siRNA vector, MDA-MB-231/LKB1, and MDA-MB-231/siRNA LKB1 cells. (**B)** Representative quantitation from three independent experiments. The values of MDA-MB-231 are expressed relative to the respective controls (β-actin), which were given an arbitrary value of 1. Bars, SE. *p<0.05, #p<0.05.

**Figure 4:**
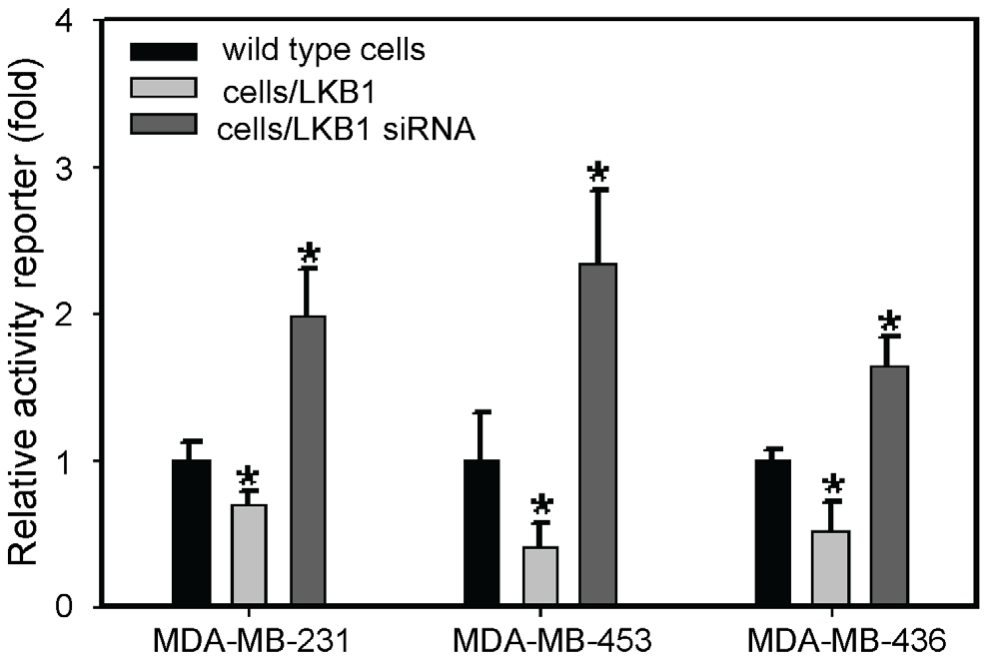
The transcriptional activity of Hh pathway in MDA-MB-231, MDA-MB-453,MDA-MB-436 detected by Gli reporter assay. Silencing of LKB1 resulted in a significant increase of the transcriptional activity, compared with the wild type. * P<0.05, And overexpression of LKB1 resulted in a significant decrease of the transcriptional activity, compared with the wild type. * P<0.05, Student’s t-test.

To study the potential effects of LKB1 on the Hh signaling pathway, we examined the expression of Hh signaling molecules, including Shh, Smo, GLI1, Sufu, Ptch, and Hip, by RT-PCR and Western blot analysis after overexpression or knockdown of LKB1. The mRNA and protein levels of Shh, GLI1 and Smo (P<0.05) were significantly reduced in LKB1-transfected MDA-MB-231 cells, and high levels of Shh, GLI1, and Smo expression (P<0.05) were detected in siRNA LKB1-transfected MDA-MB-231 cells. Meanwhile, the expressions of Sufu and Hip was increased and decreased respectively after overexpression and knockdown of LKB1 in MDA-MB-231 cells (P<0.05). Also, we did not observe significant changes in PTCH expression in two of these transfected cell preparations (Figure 2 and 3). Furthermore, we detected the effets of LKB1 on hedgehog activity using Gli-reporter assay in MDA-MB-231, MDA-MB-436 and MDA-MB-453 cells. We found that negative correlation between LKB1 and glioma-associated oncogene homologue 1 (GLI1) in these three breast cancer cell lines (Figure 4).

### Knockdown of LKB1 Enhance the Tumor Growth in the Mammary Fat Pad of Nude Mice

**Figure 5:**
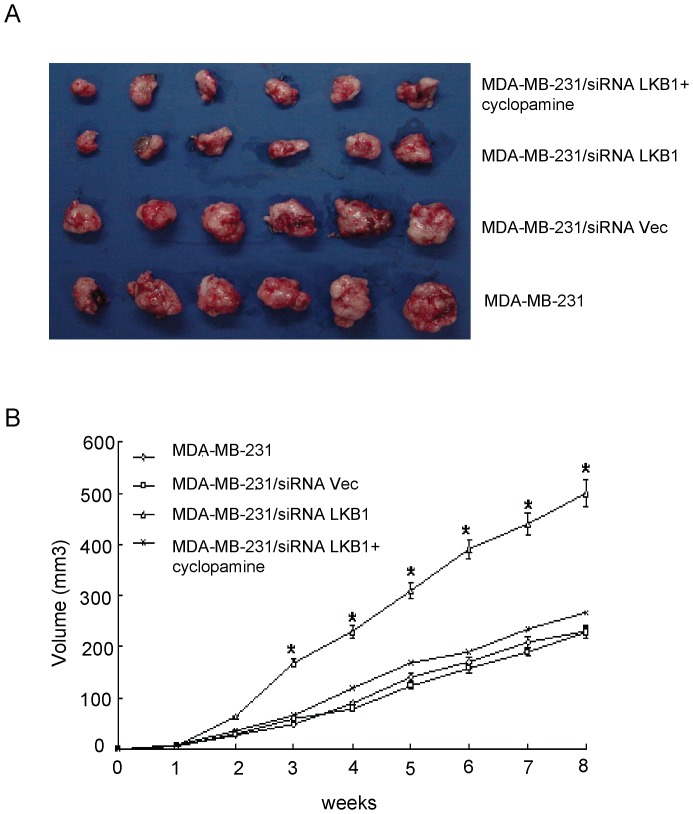
Knockdown of LKB1 Expression Significantly Increased the Growth of MDA-MB-231 xenograft growth in nude mice. (**A**) Tumor specimens dissected from the nude mice xenografted with different MDA-MB-231 cells. (**B**) Tumor growth curve. The data were generated from six mice in four different groups. *P<0.05, as compared with control groups.

**Figure 6:**
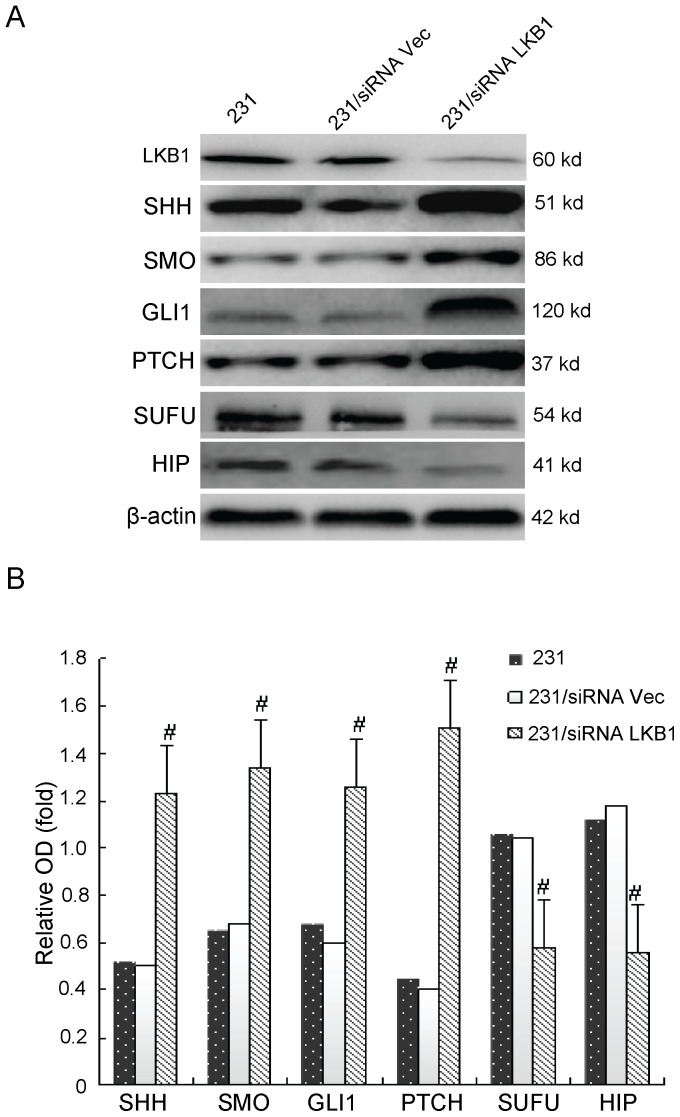
The relative protein levels of LKB1, SHH, SMO, GLI1, PTCH, SUFU, and HIP, which were detected by Western blotting (A) in MDA-MB-231, MDA-MB-231/siRNA vector, and MDA-MB-231/siRNA LKB1 xenografts. (**B**) Representative quantitation from three independent experiments. The values of MDA-MB-231 are expressed relative to the respective controls (β-actin), which were given an arbitrary value of 1. Bars, SE. #p<0.05.

Our previous work has shown that overexpression of LKB1 significantly reduced the tumor growth in nude mice. In this study, we injected siRNA LKB1-transfected MDA-MB-231 cells into the mammary fat pad of nude mice to assess the effect of LKB1 knockdown on the tumor growth in vivo. As expected, LKB1-transfected siRNA MDA-MB-231 cells grew much faster than either the mock-transfected or wild-type cells in nude mice (P<0.05**)** (Figure 5). Similar effects of LKB1 knockdown on the protein expression of Hh signaling molecules in vivo were also observed as shown in Figure 6. Meahwhile, administration of cyclopamine reversed LKB1 siRNA-transfected MDA-MB-231-induced tumor growth (Figure 5).

### Effects of Cyclopamine on Cell Cycle and Cell Apoptosis

**Figure 7:**
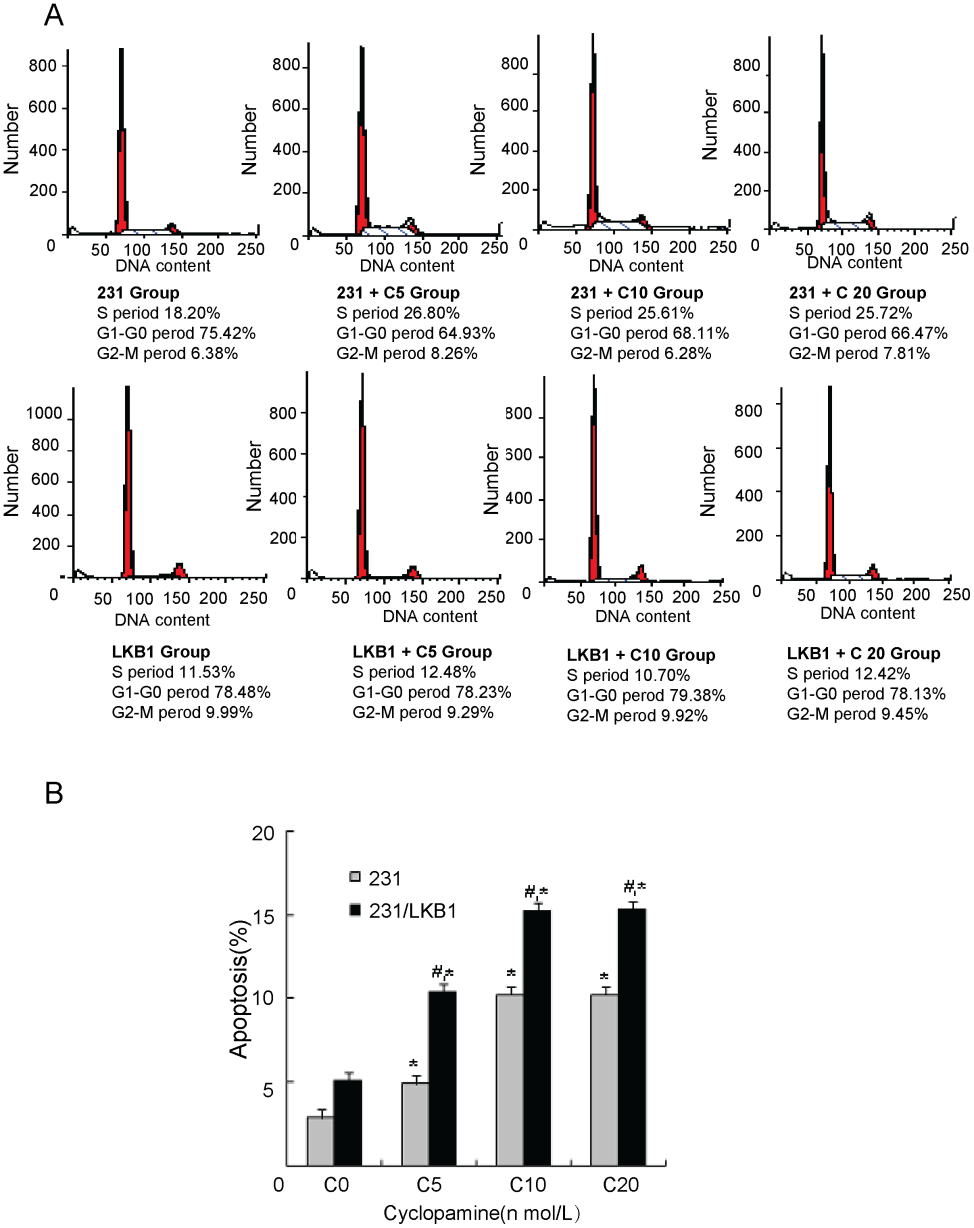
Effects of cyclopamine on the cell cycle and apoptosis of LKB1-transfected MDA-MB-231 cells. The cells were harvested after 48 h of cyclopamine treatment in four different concentrations (0 nmol/L, 5 nmol/L, 10 nmol/L and 20 nmol/L). The cells were stained with BrdU or propidium iodide. Distribution of the cell cycle (**A**) and the rate of apoptosis (**B**) were determined using flow cytometry. C0, 0 nmol/L; C5, 5 nmol/L; C10, 10 nmol/L; C20, 20 nmol/L.

To investigate the influence of cyclopamine, a specific inhibitor of sonic hedgehog (Shh), on early apoptosis of MDA-MB-231 after LKB1 siRNA transfection, we examined the apoptosis of cells using cytoflow analysis. As shown in Figure 7, cyclopamine (0-20 nmol/L) dose-dependently (P<0.05) increase the cell apoptosis in both control and LKB1 siRNA transfected cells. Furthermore, the apoptosis rate of LKB1 siRNA-transfected group was significantly increased in comparison with MDA-MB-231 control group respond to different concentrations of cyclopamine (0-20 nmol/L) (P<0.05).

### The Expression of LKB1 and Hh in Breast Cancer Specimens

**Figure 8:**
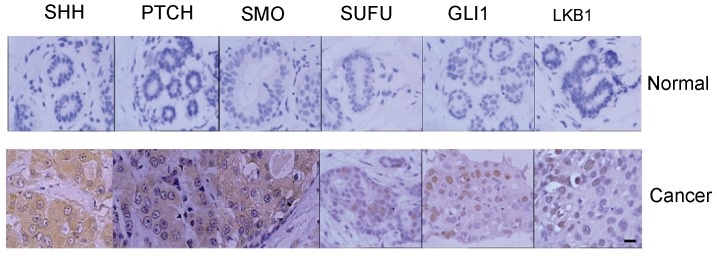
Immunohistochemical staining of GLI1, Ptch, Smo, Sufu, Shh, and LKB1 from normal and neoplastic breast tissues. Representative normal and tumor samples show an obvious correlation between GLI1, Ptch, Smo, Shh, Sufu, and LKB1 staining.

To further clarify the correlation of LKB1 and Hh signaling molecules in human breast cancer development, we analysis the correlation of these signaling molecules with LKB1 in 75 breast cancer specimens by immunohistochemical staining. The characteristics of the study subjects were showed as Table 2. Our results showed that 69 (92.0%), 58 (77.3%), 65 (86.7%), 50 (66.7%), 34 (45.3%) and 33 (44.0%) of specimens are positively stained for Shh, GLI1, Smo, Ptch, Sufu and LKB1, respectively. Moreover, we found that the levels of the Shh, GLI1, Smo, Sufu negatively correlated with the LKB1, respectively. (*p*<0.05, Spearman’s rank test). Meanwhile, positive correlation was demonstrated between Sufu and LKB1 protein expression (*p*<0.05). There was no significant correlation between ptch and LKB1 protein expression in these specimens (p>0.05, Table 3, Fig. 8).

**Table 2:** Characteristics of the study subjects.

	No. of Patients(*N* = 75)
**Age (yrs.)**	
**Median**	**48.5**
**Range**	**28-68**
**<40 years**	**20**
**>40 years**	**55**
**Surgical treatment**	
**Lumpectormy**	**3**
**Lumpectormy +radiotherapy**	**20**
**Mastectormy**	**42**
**Tumor size (cm)**	
**<3 cm**	**66**
**>3 cm**	**9**
**Node status**	
**Negative**	**41**
**Positive**	**34**
**Histological type**	
**Ductal**	**61**
**Lobular**	**4**
**Medullary**	**4**
**Paget’s disease**	**1**
**Adenocarcinoma**	**3**
**Others**	**2**

**Table 3:** Association between LKB1 expression and Hh signaling molecules.

Effector Molecules In Hedgehog Signaling		LKB1					
		–	+	++	+++	P	r
Shh	–	1	1	2	2	0.006	-0.315
	+	8	5	1	1		
	++	19	13	4	0		
	+++	14	3	1	0		
GLI1	–	6	6	3	2	0.023	-0.262
	+	19	11	2	1		
	++	8	2	3	0		
	+++	9	3	0	0		
Smo	-	2	3	3	2	0.004	-0.333
	+	16	12	1	1		
	++	18	6	4	0		
	+++	6	1	0	0		
Ptch	–	13	6	3	3	0.086	-0.199
	+	16	13	4	0		
	++	8	2	1	0		
	+++	5	1	0	0		
Sufu	–	27	9	5	0	0.014	0.284
	+	11	9	0	1		
	++	4	3	1	0		
	+++	0	1	2	2		

## Discussion

LKB1 has been implicated as a tumor suppressor gene. Deregulation of Hh signaling molecules has also been demonstrated in the pathogenesis of carcinoma[11]-[13]. Our previous study demonstrated that overexpression of LKB1 in breast cells results in the upregulation of p21WAF1/CIP1 and downregulation of cyclin D1 and cyclin E protein expression. It is well known that cyclin D1 is the target gene of Hh signaling in breast cancer [14]. Moreover, Several studies have found that PKA plays an important role in Hh signaling, which in turn determines the activity of cAMP. As LKB1 can also affect the state of cAMP [15], this raises the question of the relationship between LKB1 and Hh in human breast cancer patients.

In present study, we transfected the LKB1 gene into the MDA-MB-231 cell line with low LKB1 expression. Meanwhile, we also knocked out the LKB1 gene in the LKB1 transfected MDA-MB-231 cells. The results indicated that significant downregulation of Shh, GLI1, and Smo expression in both mRNA and protein levels in LKB1-transfected MDA-MB-231 cells and upregulation these effectors in siRNA LKB1-transfected MDA-MB-231 cells. In contrast, Hip and Sufu mRNA and protein expression levels were significantly upregulated in LKB1-transfected MDA-MB-231 cells and downregulated in siRNALKB1-transfected MDA-MB-231 cells. Furthermore, we also showed that siRNA LKB1-transfected MDA-MB-231 cells grew significantly faster than mock-transfected or wild-type cells in nude mice.

Recent studies have demonstrated that aberrant activation of the Hh cascade in human breast cancer can be caused by elevated levels of Hh ligands, such as GLI1 [16]. Thus, ligand-dependent Hh stimulation may be a key factor that promotes tumor growth. It is known that GLI1 proteins are mediators of more than the Hh signaling pathway, and overexpression of GLI1 has been reported in several types of human cancer [17], [18]. Further more, GLI1 regulates the transcription of several other genes, including genes that control platelet-derived growth factor, vascular endothelial growth factor, cyclin D, cyclin E, and Myc [19]. Meanwhile, Hip is a glycoprotein that has a high affinity for Hh and can restrain Hh through binding to its receptor [20]. Similarly, Sufu is another important regulatory protein (fused inhibitors), which also plays a key role in negatively regulating Hh signaling [21], [22]. Based on above results that LKB1 is negatively correlated with Hh signaling molecules expression, we speculated that LKB1 can affect the expression of Hh signaling molecules, which in turn involve in the regulation of breast cancer growth.

To test this speculation, cyclopamine, a specific inhibitor of Smo was used to examine whether Hh signaling pathway was involved in the effects of LKB1 on breast cancer growth. As expected, our data showed the rate of cell apoptosis dose-dependently increased respond to different concentrations of cyclopamine in both wild-type MDA-MB-231 cells and LKB1-transfected cells. Importantly, the apoptosis rate of the LKB1-transfected cells is higher than wild control, suggesting that LKB1 can lead to a significant reduction of breast cancer cells in collaboration with cyclopamine. This also implied that Hh signaling molecules could be contributed to this inhibitory action.

Finally, we examined the expression of LKB1 and Hh in 75 cases of human breast cancer samples. Consistent with the results derived from cell line studies, the expression of the Hh signaling molecules (Shh, GLI1, Smo, Sufu) was found to be negatively correlated with LKB1 expression in human breast cancer specimens. The activating Hh signaling pathway in human breast cancers does not seem to be related to the tumor size but may be related to the age, molecular subtypes and lymphoid node metastasis. Our previous study also showed that low expression of the LKB1 protein in human breast cancer is significantly associated with decreased survival.

In conclusion, our data support the hypothesis that LKB1 may inhibit tumorigenesis by regulating Hh signaling in breast cancer. Hh signaling molecules are only expressed in breast cancer, and their expression is correlated with a reduced expression of LKB1. The regulatory relationship between LKB1 and Hh signaling pathway may have role in breast cancer prognosis and treatment.
